# Ion‐scale secondary flux ropes generated by magnetopause reconnection as resolved by MMS

**DOI:** 10.1002/2016GL068747

**Published:** 2016-05-18

**Authors:** J. P. Eastwood, T. D. Phan, P. A. Cassak, D. J. Gershman, C. Haggerty, K. Malakit, M. A. Shay, R. Mistry, M. Øieroset, C. T. Russell, J. A. Slavin, M. R. Argall, L. A. Avanov, J. L. Burch, L. J. Chen, J. C. Dorelli, R. E. Ergun, B. L. Giles, Y. Khotyaintsev, B. Lavraud, P. A. Lindqvist, T. E. Moore, R. Nakamura, W. Paterson, C. Pollock, R. J. Strangeway, R. B. Torbert, S. Wang

**Affiliations:** ^1^ Blackett LaboratoryImperial College LondonLondonUK; ^2^ Space Sciences LaboratoryUniversity of CaliforniaBerkeleyCaliforniaUSA; ^3^ Department of Physics and AstronomyWest Virginia UniversityMorgantownWest VirginiaUSA; ^4^ NASA Goddard Space Flight CenterGreenbeltMarylandUSA; ^5^ Department of AstronomyUniversity of MarylandCollege ParkMarylandUSA; ^6^ Department of Physics and AstronomyUniversity of DelawareNewarkDelawareUSA; ^7^ Department of PhysicsMahidol UniversityBangkokThailand; ^8^ Department of Earth, Planetary and Space SciencesUniversity of CaliforniaLos AngelesCaliforniaUSA; ^9^ Department of Climate and Space Sciences and EngineeringUniversity of MichiganAnn ArborMichiganUSA; ^10^ Institute for the Study of Earth, Oceans and SpaceUniversity of New HampshireDurhamNew HampshireUSA; ^11^ Southwest Research InstituteSan AntonioTexasUSA; ^12^ Laboratory for Atmospheric and Space PhysicsUniversity of Colorado BoulderBoulderColoradoUSA; ^13^ Swedish Institute of Space PhysicsUppsalaSweden; ^14^ Institut de Recherche en Astrophysique et PlanétologieUniversité de ToulouseToulouseFrance; ^15^ Centre National de la Recherche Scientifique, UMR 5277ToulouseFrance; ^16^ School of Electrical EngineeringRoyal Institute of TechnologyStockholmSweden; ^17^ Space Research InstituteAustrian Academy of SciencesGrazAustria; ^18^ Denali ScientificHealyAlaskaUSA

**Keywords:** magnetic reconnection, magnetopause, flux rope, secondary island, magnetospheric multiscale

## Abstract

New Magnetospheric Multiscale (MMS) observations of small‐scale (~7 ion inertial length radius) flux transfer events (FTEs) at the dayside magnetopause are reported. The 10 km MMS tetrahedron size enables their structure and properties to be calculated using a variety of multispacecraft techniques, allowing them to be identified as flux ropes, whose flux content is small (~22 kWb). The current density, calculated using plasma and magnetic field measurements independently, is found to be filamentary. Intercomparison of the plasma moments with electric and magnetic field measurements reveals structured non‐frozen‐in ion behavior. The data are further compared with a particle‐in‐cell simulation. It is concluded that these small‐scale flux ropes, which are not seen to be growing, represent a distinct class of FTE which is generated on the magnetopause by secondary reconnection.

## Key Points

- Ion‐scale flux ropes are observed during magnetopause reconnection
- The largely force‐free flux ropes exhibit filamentary currents and nonideal ion behavior
- Small flux content and comparison with simulation indicate a secondary reconnection origin

## 1 Introduction

The dayside magnetopause is an important location for studying magnetic reconnection in situ. Flux transfer events, typically identified as a bipolar signature in the component of the magnetic field normal to the magnetopause [*Russell and Elphic*, 1978], are a commonly observed feature of magnetopause reconnection [e.g., *Rijnbeek et al*., 1984; *Wang et al*., 2005, 2006; *Kawano and Russell*, 1997; *Fear et al*., 2008, 2009; *Zhang et al*., 2012]. Several competing models of flux transfer event (FTE) formation have been developed to date, based on transient/temporally varying reconnection [*Scholer*, 1988; *Southwood et al*., 1988], multiple, and possibly sequential, *X*‐line reconnection [*Lee and Fu*, 1985; *Raeder*, 2006], and spatially limited reconnection [*Russell and Elphic*, 1978].

Experimental analysis shows that FTEs are often more accurately described as flux ropes [e.g., *Xiao et al*., 2004; *Eastwood et al*., 2012]. The reported flux content of these structures is of the order of 1–10 MWb [e.g., *Rijnbeek et al*., 1984; *Hasegawa et al*., 2006], with sizes of the order of 1 to a few Earth radii (*R_E_*). More generally, flux ropes, and ion‐scale flux ropes in particular, are an important component of reconnection‐driven particle acceleration models [*Drake et al*., 2006a; *Chen et al*., 2008], arise naturally in 2‐D and fully 3‐D simulations of reconnection [*Drake et al*., 2006b; *Daughton et al*., 2011a, 2011b], and may even also modulate the reconnection rate itself [*Karimabadi et al*., 2007].

Efforts to understand the physics of magnetopause flux ropes and their internal structure have, to some extent, been somewhat limited by the time resolution of the available measurements, particularly plasma moments measured with a cadence of several seconds which may be comparable to the duration of some flux rope observations. Furthermore, insufficiently resolved observations may place an artificially high limit on the minimum flux rope size and thus impede attempts to accurately determine their size distribution [*Fermo et al*., 2011].

Here we present novel measurements from the four‐spacecraft Magnetospheric Multiscale (MMS) mission [*Burch et al*., 2015] of two flux ropes observed sequentially in a magnetopause reconnection jet. These flux ropes are small (radius ~ 7 magnetosheath ion inertial lengths (*d*_i_) ~ 0.17 *R_E_*) with duration of a few seconds in the data. The MMS tetrahedron was at 10 km (~0.14*d*_i_) scale, and the multipoint observations are used, in combination with computer simulation, to study their size, current density filamentation and structure, and nonideal plasma behavior.

## 2 Observations

**Figure 1:**
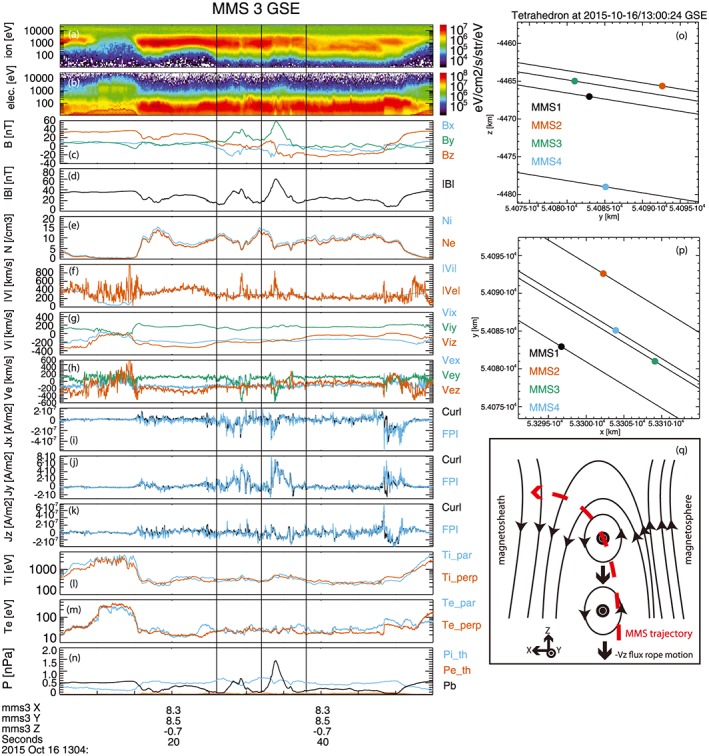
(a–n) Time series of ion and electron spectrograms, magnetic field components, and strength, density, speed, ion and electron velocity, current density, and ion and electron temperature; (o, p) MMS tetrahedron configuration; and (q) cartoon of encounter. Data are shown in GSE.

The MMS observations were made on 16 October 2015, 13:04:05 UT to 13:04:55 UT, shortly before MMS encountered the electron dissipation region at 13:07:02 UT [*Burch et al*., 2016]. Figures 1a–1n present MMS3 flux gate magnetometer (FGM) [*Russell et al*., 2014] and fast plasma experiment (FPI) [*Pollock et al*., 2016] observations using Geocentric Solar Ecliptic (GSE) coordinates. On this scale, data from the individual satellites appear identical. The data were captured at high time resolution: FPI moments are constructed from all‐sky electron and ion distributions at 30 ms and 150 ms cadence, respectively.

Figures 1o and 1p show that MMS was located duskward of the Sun‐Earth line, at [8.3, 8.5, −0.7] *R_E_*. The tetrahedron size was ~10 km, with MMS1, MMS2, and MMS3 approximately forming an equilateral triangle confined to the *x*_GSE_‐*y*_GSE_ plane and MMS4 located below this plane at smaller values of *z*_GSE_. MMS was initially located in the magnetosphere (low density, northward magnetic field) and started to cross the magnetopause at 13:04:15 UT (when the plasma density began to increase, and *B_z_* decrease), eventually reaching the magnetosheath flow proper at 13:04:40 UT. During this crossing, an enhanced flow was observed in the −*v_z_* direction when compared to the later magnetosheath interval, and MMS encountered two flux transfer events. These events are marked by vertical lines corresponding to the interval 13:04:26 UT to 13:04:32 UT (event #1) and 13:04:32 UT to 13:04:38 UT (event #2). They are identified more specifically as flux ropes (“FR#1” and “FR#2”) because of the correlated increase in |*B*| and *B_y_*, centered on the bipolar (negative/positive) signature in *B_x_*.

**Figure 2:**
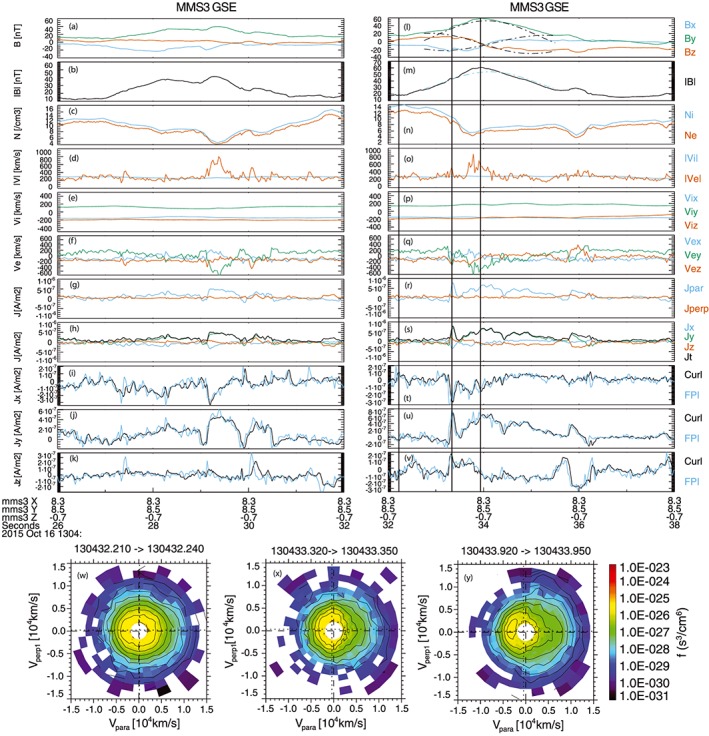
(a–k) Time series of magnetic field components and strength, density, speed, ion and electron velocity, and current density for the first flux rope event in GSE; (l–v) same data products for the second flux rope event; (w–y) electron distribution function cuts in the *v*_para_/*v*_perp_ plane at times corresponding to the vertical lines in Figures 2l–2v.

Figure 1q provides a summary of the observations and their context. The red dashed line shows the trajectory of MMS relative to the magnetopause and the flux ropes. Initially located on the magnetospheric side, MMS skirted the magnetospheric edge of the first flux rope, since *B_z_* was small but remained positive. During the encounter with the second flux rope, a bipolar signature in *B_z_* was observed in conjunction with the bipolar signature in *B_x_*. Furthermore, these reversals are coincident with the peak in |*B*| and *B_y_*, indicating that when crossing the flux rope MMS cut through the center of the structure. The sense of the bipolar signatures in both events is consistent with southward flux rope motion, confirming that the two flux ropes are observed sequentially. The trajectory of the second event allows a force‐free flux rope model [e.g., *Eastwood et al*., 2012, and references therein] to be applied (time interval 13:04:32.75 UT to 13:04:35.5 UT) which gives an axis orientation of [−0.012, 0.989, −0.149] GSE and provides reasonable qualitative agreement to the features in the magnetic field time series (Figure 2l).

Four‐spacecraft timing analysis [e.g., *Schwartz*, 1998] can also be applied to gain quantitative information about the orientation and motion of these structures relative to the spacecraft. In the case of a cylindrically symmetric flux rope, it can be shown that the times at which each spacecraft observes the peak field strength define a plane which is perpendicular to the tetrahedron direction of motion (in the flux rope frame) and contains the axis of the flux rope. The timing analysis result thus defines the orientation of the spacecraft trajectory through the flux rope in the plane perpendicular to the flux rope axis. Consequently, the perpendicular motion of the flux rope axis relative to the tetrahedron can be computed.

FR#1 is found to be moving at *v*_1_ ~ 223 km s^−1^ along **n_1_** = [−0.375, −0.128, −0.918] GSE relative to MMS. FR#2 is found to be moving at *v*_2_ ~ 264 km s^−1^ along **n_2_** = [−0.784, −0.075, −0.617] GSE relative to MMS. The motion of both is consistent with the features of the time series in Figures 1a–1m, since MMS crossed from the leading edge of the flux rope on the magnetospheric side to the trailing edge of the flux rope on the magnetosheath side. Consequently, from the perspective of MMS the flux rope appeared to move in the −*x*_GSE_ and −*z*_GSE_ directions. The velocity components along *x*_GSE_ and *z*_GSE_ are *v*_2,*x*_ ~ −207 km/s and *v*_2,*z*_ ~ −162 km/s. Thus, FR#2 is moving slightly more slowly than FR#1 along the magnetopause in the −*z*_GSE_ direction.

These results, together with flux rope fitting (Figure 2l) showing the axis of the second flux rope pointing predominantly in the +*y*_GSE_ direction, indicate that the GSE coordinate system is sufficient to understand the main features of the data shown in Figure 1. The duration of FR#1 (corresponding to the interval of enhanced |*B*| from 13:04:27 to 13:04:31) is ~4 s. The length of the chord through FR#1 is estimated to be ~ 890 km ~ 12*d*_i_ since the magnetosheath density ~ 9.8 cm^−3^ with a corresponding ion inertial length *d*_i_ = 73 km. Regarding FR#2, the fact that *B_x_* and *B_z_* reversed sign at the time that |*B*| peaked is evidence that MMS passed very close to its central axis. The duration of FR#2 (13:04:32.4–13:04:36.6) thus corresponds to a diameter of ~1100 km ~ 15*d*_i_. Both flux ropes are therefore of similar size. Finally, we note that the reconnection jet speed in the −*z*_GSE_ direction is comparable to the timing‐derived speeds, and there is no evidence for converging flow in the *z*_GSE_ direction across each island. Such observations (which are rare) would be associated with an “active” flux rope [*Hasegawa et al*., 2010; *Øieroset et al*., 2011, 2014].

## 3 Current Density Structure and Filamentation

The current density can be calculated using the curlometer technique [e.g., *Robert et al*., 1998; *Dunlop et al*., 2002], which assumes that the magnetic field varies linearly across the spacecraft tetrahedron. It is best suited to investigating phenomena where the scale size of the tetrahedron is much less than the structure under consideration. This criterion is well satisfied here, as the spacecraft separation is more than an order of magnitude less than the structure size. The high quality of the FPI data is such that the current density can also be calculated directly according to the formula **J** = *n*_e_ *e*(**v_i_ − v_e_**) where *n*_e_ is the electron number density, *e* is the elementary coulomb charge, and **v_i_** and **v_e_** are the ion and electron velocities (we assume a quasi‐neutral proton plasma). Here the ion moments are interpolated onto the electron moments to compute **J** at the highest possible time resolution.

Extremely good agreement between both techniques is found over the whole interval (Figures 1i–1k). Both methods reveal that there is considerable structure and variability in the current density time series. This is not immediately evident from the magnetic field time series itself, which appears rather smooth. The very good agreement between the two independent methods confirms that these features are physical. Current densities calculated using particle data from the other three satellites show similarly good agreement.

Figure 2 shows the current density in each flux rope in more detail. The components of the current density show that the current is also predominantly in the +*y*_GSE_ direction. As a more general observation, in FR#1, the peak current density is smaller compared to that in FR#2. This is most probably associated with the different trajectory that MMS took through each structure. The agreement between the two methods of calculating the current density is excellent. This validates the interpolation of the ion data, and the comparison reveals a fundamental feature of the plasma now resolved by MMS: the ion velocity time series is indeed considerably smoother and less variable than the electron velocity time series; this is not simply because it is sampled at lower time resolution. Note that the FPI current density is calculated at the vertex of the tetrahedron (here at MMS3), whereas the curlometer represents an “average” current density. The fact that MMS3 is north of the tetrahedron barycenter means that the FPI current time series tends to lead the curlometer time series, as the structures are moving from north to south. In fact, there is evidence that in places the current density gradients are sharper in the FPI data than in the curlometer. This may indicate that there is current density structure on scales below the tetrahedron scale.

Figures 2g and 2r show the curlometer‐derived current density within each flux rope parallel and perpendicular to the magnetic field. It is clear that the current density, which is carried by the electrons, is predominantly field aligned. Correspondingly, the **J × B** force is found to be small, and this directly confirms the validity of the force‐free model as applied to FR#2. An interesting experimental observation is that the thermal pressure is not negligible (Figure 1n), and superthermal electrons are present in the flux ropes (Figure 1b). However, the thermal pressure is relatively uniform and isotropic throughout. This demonstrates that force‐free solutions are not restricted to low *β* plasmas.

We take advantage of FR#2's largely force‐free nature to estimate its flux content using an analytic formula [*Eastwood et al*., 2012]. Here for a model peak field strength of 54.4 nT and a radius of 550 km, the flux content is Φ = 0.4158[2π*B*_0_ *R*_f_^2^ *J*_1_(2.40482)] = 22.3 kWb. This is considerably less than the typically observed FTE flux content of ~ 1 MWb [e.g., *Rijnbeek et al*., 1984; *Hasegawa et al*., 2006].

We now examine the features of the current density time series more closely. Within FR#1, the current density in the leading edge is initially relatively weak and reduces to nearly zero at 13:04:29.1 UT. The current density then immediately increases and is enhanced over the interval where |*B*| is maximized until 13:04:29.9 UT where it again reduces to close to zero. A third enhancement in current density then occurs which persists until 13:04:30.5 UT. In FR#2, there is an initial channel of strong parallel current of duration ~0.15 s which peaks at 13:04:33.35 UT. This current filament is on the “magnetospheric” edge of the flux rope. The duration corresponds to a thickness of 40 km or 0.54*d*_i_. The current density then reduces to close to zero, before increasing to a peak just after 13:04:34 UT which corresponds to the maximum field strength in the core of the flux rope. The current density then falls to zero at just after 13:04:34.4 UT before increasing and then decreasing again. A further current filament is seen between 13:04:35.75 and 13:04:36.3 UT which sits near the “magnetosheath” edge of the flux rope.

The electron populations that carry these currents are resolved by MMS. For example, Figures 2w–2y show three examples of electron distributions captured before and during FR#2, presented in the *v*_para_/*v*_perp_ plane, and measured at 30 ms cadence with the midtime corresponding to the vertical lines in Figures 2l–2v. Whereas the distribution in Figure 2w is taken at a quiet time before FR#2 to provide context, in both the current filament and at the center of the flux rope (Figures 2x and 2y) the current is carried by a relatively cold population moving antiparallel to the magnetic field. There is also the signature of a hotter population moving parallel to the magnetic field, which indicates the presence of magnetospheric plasma and is consistent with the expected 3‐D topology of magnetopause flux ropes.

## 4 Nonideal Plasma Behavior

**Figure 3:**
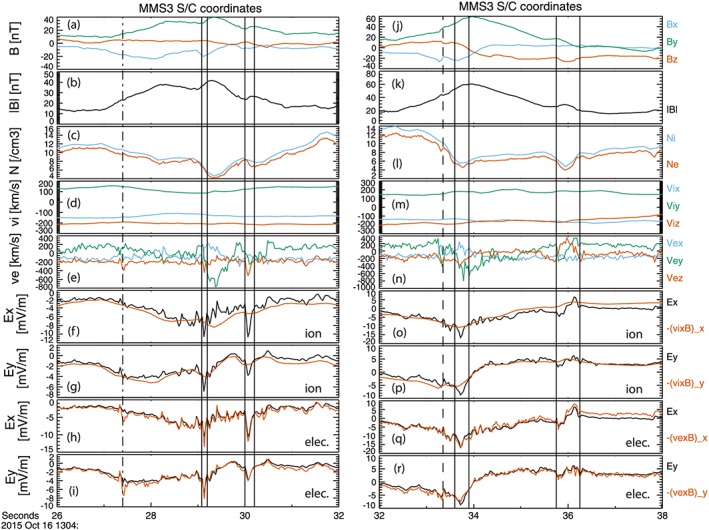
(a–i) MMS3 time series of magnetic field components and strength, density, ion and electron velocity, and spin plane electric field; (j–r) same data products for the second flux rope event. Data are shown in spacecraft coordinate system.

In both these flux rope events, there is predominantly parallel current. We now examine evidence for nonideal behavior associated with differential perpendicular motion, that is, deviations from **E** + **v_i,e_** × **B** = 0. Note that a deviation in only one component is required for the vector **E** + **v** × **B** to be nonzero. Figure 3 compares data from the spin plane electric field double probes (shown in black) [*Lindqvist et al*., 2014] with −**v_i_** × **B** and −**v_e_** × **B** (shown in red). The data are presented in the spacecraft coordinate system, which is close to GSE. Although only MMS3 data are shown, it should be emphasized that the same features are visible on all four MMS satellites. Overall, there is good agreement between the time series (disregarding small systematic constant offsets), but there are intervals where there is significant deviation in the ion plasma, whereas the electron plasma remains frozen in.

For example, in FR#1, such deviations are seen at 13:04:29.1–13:04:29.2 UT and 13:04:30.0–13:04:30.2 UT (marked by vertical lines). These intervals are adjacent to the enhanced central current density channel in FR#1. Throughout the flux rope *E_x_* is negative. During both deviations *E_x_* is more negative than −(**v_i_** × **B**)*_x_* and so since the magnetic field points predominantly in the +*B_y_* direction, this indicates that the ions are moving more slowly than the electrons in the axial direction (~ −*z*_GSE_) direction. In FR#2 a first deviation in ion behavior is seen at 13:04:33.6–13:04:33.9 UT. This deviation occurs after the strong parallel filamentary current at the magnetospheric edge of the flux rope (dashed line). The first interval of non‐frozen‐in ion behavior is therefore inside the flux rope. Again, *E_x_* is negative, and *E_x_* is more negative than −(**v_i_** × **B**)*_x_*, so this similarly indicates that the ions are moving more slowly. A second deviation is seen at 13:04:35.75–13:04:36.25 UT. There is a negative/positive bipolar signature in *E_x_*. This deviation is at the same time, and of similar duration, as the current filament on the magnetosheath edge of the flux rope, and so this motion is associated with the small perpendicular current observed there (Figure 2r).

In contrast to the ions, the agreement between *E*_*x*,*y*_ and (−**v_e_** × **B)**_*x*,*y*_ is extremely good throughout both events, showing that the electrons are largely frozen in throughout the encounter with both flux ropes. However, there is tentative evidence for non‐frozen‐in electron behavior, based on the deviation in *E*_*x*,*y*_ at 13:04:27.4 UT (dash‐dotted line). This will be the subject of future investigation.

## 5 Comparison With Simulations

**Figure 4:**
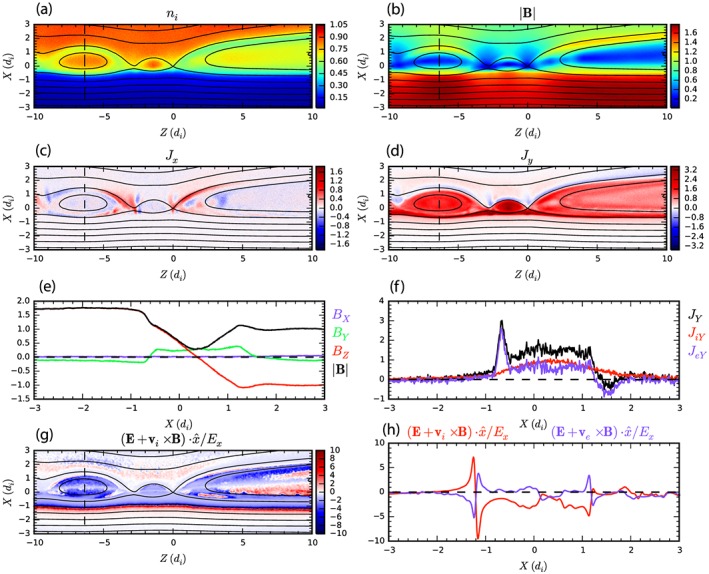
Particle‐in‐cell simulation of reconnection with cuts through a flux rope at *z* = −6.35. See text for details.

To better understand the MMS data, and FR#2 in particular, Figure 4 shows the results of a particle‐in‐cell simulation of reconnection performed using the P3D code [*Zeiler et al*., 2002], described in the supporting information.

Figures 4a and 4b show the number density and the magnetic field strength. The magnetosheath (high density, low magnetic field strength) is above the current sheet where the magnetic field points to the left (*B_z_* < 0) and the “magnetosphere” (low density, high magnetic field strength) is below where the magnetic field points to the right (i.e., *B_z_* > 0). We focus on the flux rope that has formed on the current sheet between *z* = −4 and *z* = −10. This flux rope, comparable in size to those observed by MMS, is moving to the left as the simulation evolves (equivalent to moving southward along the −*z*_GSE_ direction).

Figures 4c and 4d show maps of the *J_x_* and *J_y_* current density (red: positive, blue: negative). *J_y_*, the out‐of‐plane current density, is enhanced within the island but stronger on the magnetospheric edge. *J_x_* is enhanced around the edge of the island. Figure 4e shows the magnetic field profile along a vertical cut at *z* = −6.35. In this plot the magnetosphere is on the left‐hand side. Figure 4f shows the profile of *J_y_* total (black), ion current density *J_iy_* (red), and electron current density *J_ey_* (blue). On the magnetospheric edge there is a spike (thickness ~0.4) in the current density carried by the electrons whose magnitude, converted to SI units, is 810 nAm^−2^, which is very similar to that observed (Figure 2s). Further analysis shows that this current spike is largely field aligned. This feature is identified as the counterpart of the current spike at the start of FR #2 at 13:04:33.35 UT.

Referring more closely to this feature in Figure 2s, it can be seen that there is also a component of the current in the −*x*_GSE_ direction. This is on the magnetospheric side of the flux rope near its leading edge where *B*_*x*GSE_ is negative and corresponds to the blue region at the bottom left edge of the flux rope in Figure 4c. Figure 4e shows that *B_y_* > 0 inside the simulated flux rope, and so *J_x_* is expected to be negative to the left of the flux rope center and positive to the right which is observed in Figure 4c. We therefore conclude that the filamentary current observed by MMS at the leading edge of FR#2 corresponds to the thin parallel current layer that forms in the simulation at *x* = −0.6 in Figures 4d and 4f.

Finally, Figure 4g shows (**E** + **v_i_** × **B**)*_x_*/*E_x_*. This immediately reveals that there are regions of non‐frozen‐in ion flow confined within the flux rope and that this is not homogenous. This is confirmed by Figure 4h which is a cut along *z* = −6.35 showing the nature of the frozen in ion (red) and electron (blue) flow. The ions are not frozen in, except for a region near the center of the flux rope. This is qualitatively consistent with the MMS observations.

## 6 Discussion and Conclusions

The MMS observations reveal in new detail the existence of ion‐scale flux rope FTEs entrained in a magnetopause reconnection jet. The current, carried by the electrons, is largely parallel, and so the overall structure is well described by a relatively simple force‐free model. However, multispacecraft analysis reveals that the flux rope current density is highly structured with filament thicknesses that can be comparable to the ion inertial length. There are also regions of nonideal ion flow adjacent to these current layers and interior to the flux ropes. The second event was more conducive to comparison with simulations because of the MMS trajectory through the flux rope center. The simulation is 2.5‐D but nevertheless reproduces many of the key observed features of size, current filamentation, and non‐frozen‐in ion behavior. However, the core field is not very strongly enhanced, because of pressure balance with compressed plasma in the flux rope core that cannot escape. Also, in contrast to the observations, the simulated flux ropes are only found to be force‐free in the core region. Future comparisons with fully 3‐D simulations should even more accurately reproduce MMS observations.

FR#2 is found to have a flux content of 22.3 kWb which is considerably less than typically observed FTEs. A natural question to ask is whether these FTEs, which are small, are in the process of growing into larger structures. We do not observe a converging *v*_zGSE_ flow that would indicate the presence of active *X* lines, and thus flux being added to the structure. On the other hand, if these structures were simply to expand to typical FTE dimensions, retaining typical flux content, they would require an initial core field strength in excess of 1000 nT which is not observed. Thus, we conclude that these events are different in nature from previously reported magnetopause flux rope events. It is a well‐known feature of simulations and also magnetotail observations that flux ropes may be generated as a consequence of secondary reconnection processes at a preexisting *X* line [e.g., *Daughton et al*., 2011a]. In the simulation presented here, although the flux ropes are transient and arise not long after the initialization of the code, their formation is related to the onset of secondary instabilities in the vicinity of an *X* line and so provide insight into the structure and properties of flux ropes produced in this way.

Given the ion‐scale size, the small and stable flux content, the filamentary current structure, and the non‐frozen‐in plasma properties, together with the comparison with simulation, the analysis presented here suggests that these flux ropes are produced by secondary reconnection processes occurring in the vicinity of a single *X* line. This is not strictly the same as any of the three previously identified mechanisms discussed in section 1. Subsequent analysis of the MMS data set should reveal further information about the occurrence and statistical properties of such ion‐scale flux ropes.

## Supporting information



Supporting Information S1
