# Supplementary material for: Ion‐scale secondary flux ropes generated by magnetopause reconnection as resolved by MMS
Source: Geophys Res Lett. 2016 May 18;43(10):4716–24. doi: 10.1002/2016GL068747 (PMC5001194; doi:10.1002/2016GL068747)
Supplement: Supplementary file 1 — Supporting Information S1 [file GRL-43-4716-s001.pdf]

AGU PUBLICATIONS

Geophysical Research Letters

Supporting Information for

Ion-scale secondary flux-ropes generated by magnetopause reconnection as resolved by MMS

J. P. Eastwood,[1] T. D. Phan,[2] P. A. Cassak,[3] D. J. Gershman,[4,5] C. Haggerty,[6] K. Malakit,[7] M. A. Shay,[6] R. Mistry,[1] M. Øieroset,[2] C. T. Russell,[8] J. A. Slavin,[9] M. R. Argall,[10] L. A. Avanov,[4,5] J. L. Burch,[11] L. J. Chen,[4,5] J. C. Dorelli,[4] R. E. Ergun,[12] B. L. Giles,[4] Y. Khotyaintsev,[13] B. Lavraud,[14,15] P. A. Lindqvist,[16] T. E. Moore,[4] R. Nakamura,[17] W. Paterson,[4] C. Pollock,[18] R. J. Strangeway,[8] R. B. Torbert,[10,11] and S. Wang[4,5]

1The Blackett Laboratory, Imperial College London, London, UK
2Space Sciences Laboratory, University of California, Berkeley, CA, USA
3Dept. of Physics and Astronomy, West Virginia University, Morganstown, WV, USA
4NASA Goddard Space Flight Center, Greenbelt, MD, USA
5University of Maryland, College Park, MD, USA
6Department of Physics and Astronomy, University of Delaware, Newark, DE, USA
7Department of Physics, Mahidol University, Bangkok 10400, Thailand
8University of California, Los Angeles, Los Angeles, CA, USA
9Department of Climate and Space Sciences and Engineering, University of Michigan, Ann Arbor, MI, USA
10University of New Hampshire, Durham, NH, USA
11Southwest Research Institute, San Antonio TX, USA
12University of Colorado LASP, Boulder, Colorado, USA
13Swedish Institute of Space Physics, Uppsala, Sweden
14Institut de Recherche en Astrophysique et Planétologie, Université de Toulouse, France
15Centre National de la Recherche Scientifique, UMR 5277, Toulouse, France
16Royal Institute of Technology, Stockholm, Sweden
17Space Research Institute, Austrian Academy of Sciences, Graz, Austria
18Denali Scientific, Healy AK, USA

Supplementary information: computer simulation

The simulation was performed using the P3D code [Zeiler et al. 2002]. The simulation data are presented in a coordinate system that is directly comparable to GSE. The simulation is 2.5 dimensional $(\partial/\partial y) = 0$, and the (x,z) domain is 20.48 x 40.96 (grid scale 0.01) in distance units normalized to the magnetosheath ion inertial length. The ion to electron mass ratio is 100, and $c/V_{A,m'sheath} = 15$ where $V_{A,m'sheath}$ is the magnetosheath Alfvén speed. The time step for the particles is 0.001 times the inverse of the ion cyclotron frequency in the magnetosheath.

Initially the simulation is set up with a double hyperbolic tangent profile variation in the *x* direction of the *z* component of the magnetic field and the temperature. This corresponds to

two current sheets extending along the *z* direction at different values of *x*, contained within the domain.

The magnetic field profile across each of these current sheets is matched to a magnetospheric reconnecting magnetic field B_z = 39 nT and a reconnecting magnetosheath magnetic field B_z = 23 nT. The temperature and density profiles are set to ensure the plasma is initially in pressure balance and at rest, consistent with the magnetosheath properties (here chosen such that n = 11.3 cm^{-3} and T = 232.5 eV). Since the simulation time and length scales are normalized to the magnetosheath values, the simulation unit of length = 67.8 km, and the simulation unit of time = 2.22 s. The code and parameters are the same as those used in Burch et al. [2016].

Figure 4 in the main manuscript shows a small portion of the simulation domain at t = 16. Note that the coordinate system has been shifted, so that the current sheet lies along x = 0. Similar structure and dynamics are observed at the other current sheet (not shown).

Burch, J. L., et al. (2016), Electron-scale measurements of magnetic reconnection in space, Science, doi: 10.1126/science.aaf2939.

Zeiler, A., D. Biskamp, J. F. Drake, B. N. Rogers, and M. A. Shay (2002), Three-dimensional particle simulations of collisionless magnetic reconnection, J.Geophys.Res., 107(A9), 1230, doi:10.1029/2001JA000287.
